# Supplementary material for: Neurodevelopmental Outcome at 6 Months Following Neonatal Resuscitation in Rural Tanzania
Source: Children (Basel). 2023 May 27;10(6):957. doi: 10.3390/children10060957 (PMC10297458; doi:10.3390/children10060957)
Supplement: Supplementary file 1 [file children-10-00957-s001.zip › Supplementary information for reviewer.pdf]

Supplementary information

Table S1 Video logg for changed items:

Resuscitated (RES) or Healthy	Reason for watching video	Items changed from pass to fail	Item changed from fail to pass	MDAT DAZ* before correction	MDAT DAZ* after correction	Domain DAZ* before correction	Domain DAZ* after correction	Comment
RES	Unlikely scoring	FM 13	FM 1,2,3	-0.01	0.24	-0.83	0.68	Child refused to lay on back
RES	Unlikely scoring	FM 11		-0.93	-1.08	-1.06	-1.34	
RES	Low score	GM 7<br>FM 6		-5.50	-6.57	-2.14<br>-5.50	-7.03<br>-5.62	Possible visual and hearing impairment and focal seizures during video
RES	Understanding item scoring	GM 13		1.16	0.82	0.85	0.40	Leaning whole body at mother, not standing with support. Common way of scoring, others were not changed
RES	Unlikely score	FM 2		0.01	0.17	0.59	1.65	Well developed. Clearly following eye movements during other parts of the MDAT.
RES	Unlikely score		FM 7	-0.66	-0.33	-1.09	0.65	
RES	Unlikely score	GM 12		0.30	-0.03	0.16	-0.11	
RES	Unlikely score		FM 3,4	-0.55	-0.22	-1.80	-1.13	
Healthy	Unlikely score		FM 1,2,3	-0.57	-0.16	-0.42	1.77	Cries during FM1-3. Passes other FM items up to 13. Well developed.

**Abbreviations: MDAT; Malawi developmental assessment tool. DAZ; Development for age z-score.*

Table S2 Resuscitated included vs lost to follow up:

	Included n= 159*	Lost to follow up** n=66*	p-value
1 min Apgar < 7	75 (47.2 %)	34 (51.5%)	0.553
5 min Apgar < 7	21 (13,2%)	11 (16.7%)	0.499
Time to first ventilation in seconds (Median, IQR)	85 (75)	80 (84)	0.950
Duration of ventilation in seconds (median, IQR)	98 (140)	96.5 (138)	0.989

*Numbers from time to first ventilation and duration of ventilation were 155 included and 64 lost to follow up

** Lost to follow up included refusals, death, not located in the study area or terminated if not fulfilling inclusion crieteria

Histograms and boxplots of each MDAT domain:

Figure S1 Gross motor:

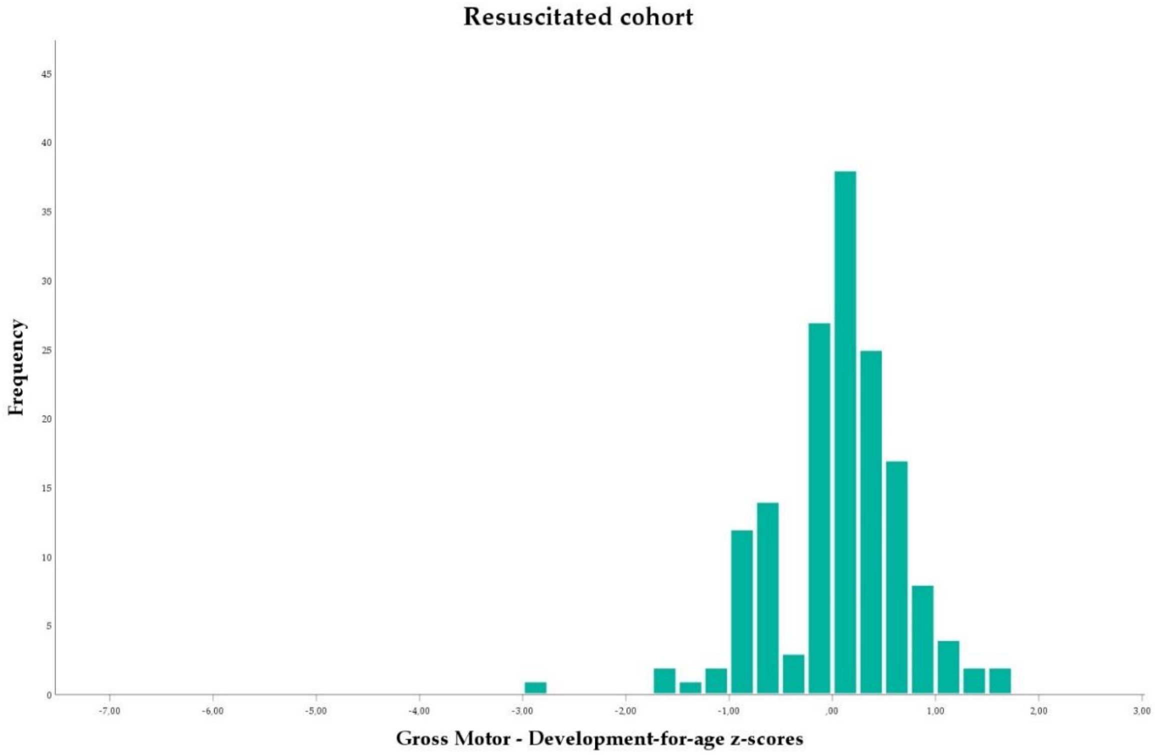

Figure S2

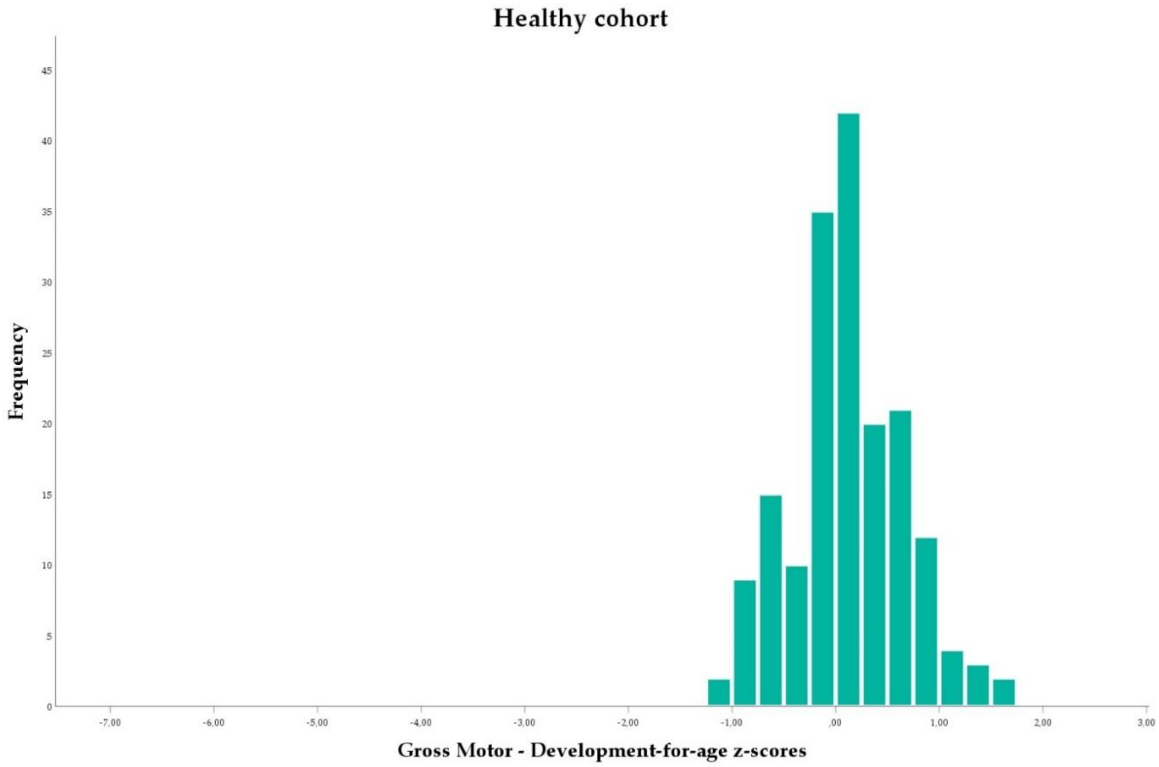

Figure S3

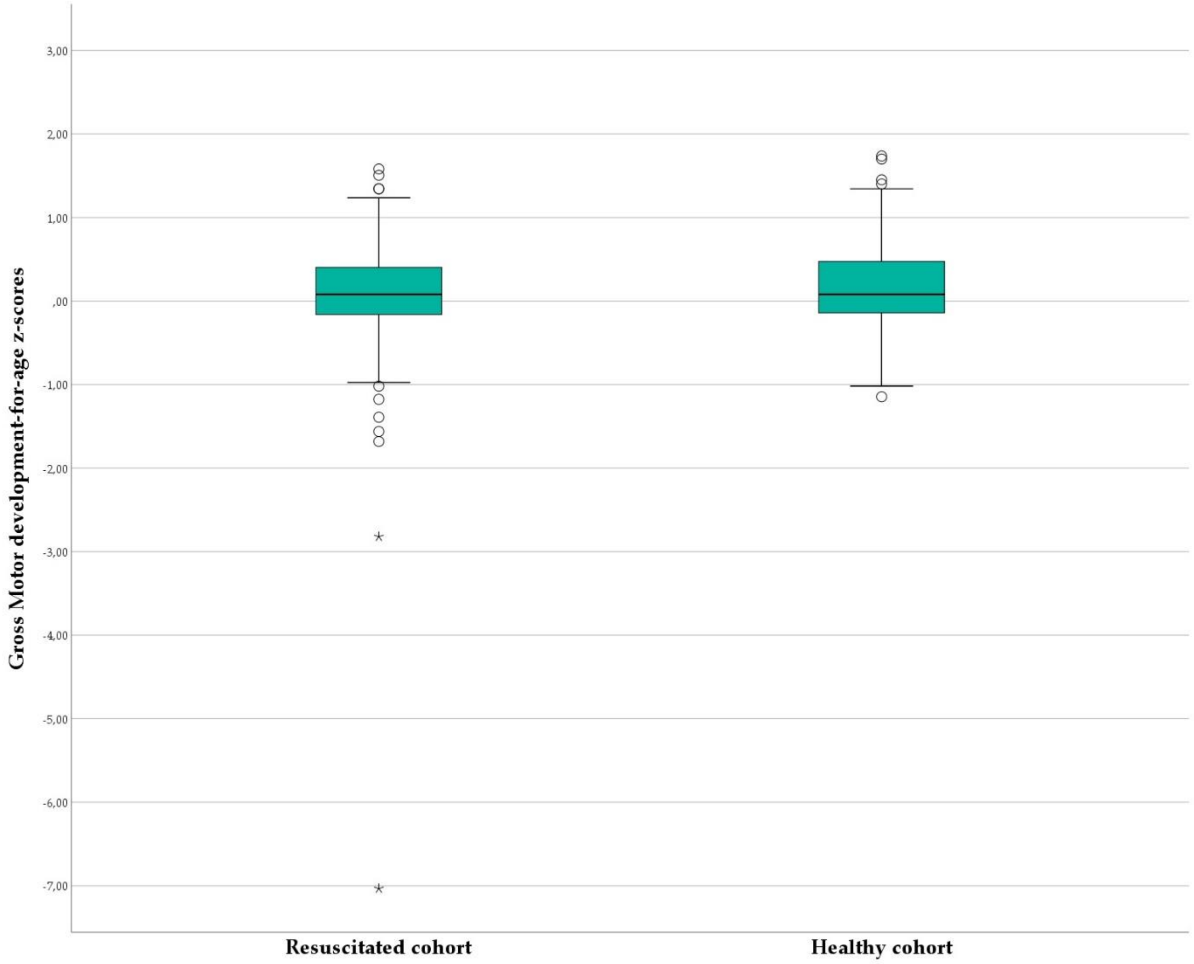
Gross Motor development-for-age z-scores
3,00
2,00
1,00
,00
-1,00
-2,00
-3,00
-4,00
-5,00
-6,00
-7,00
Resuscitated cohort
Healthy cohort

Figure S3 Fine motor:

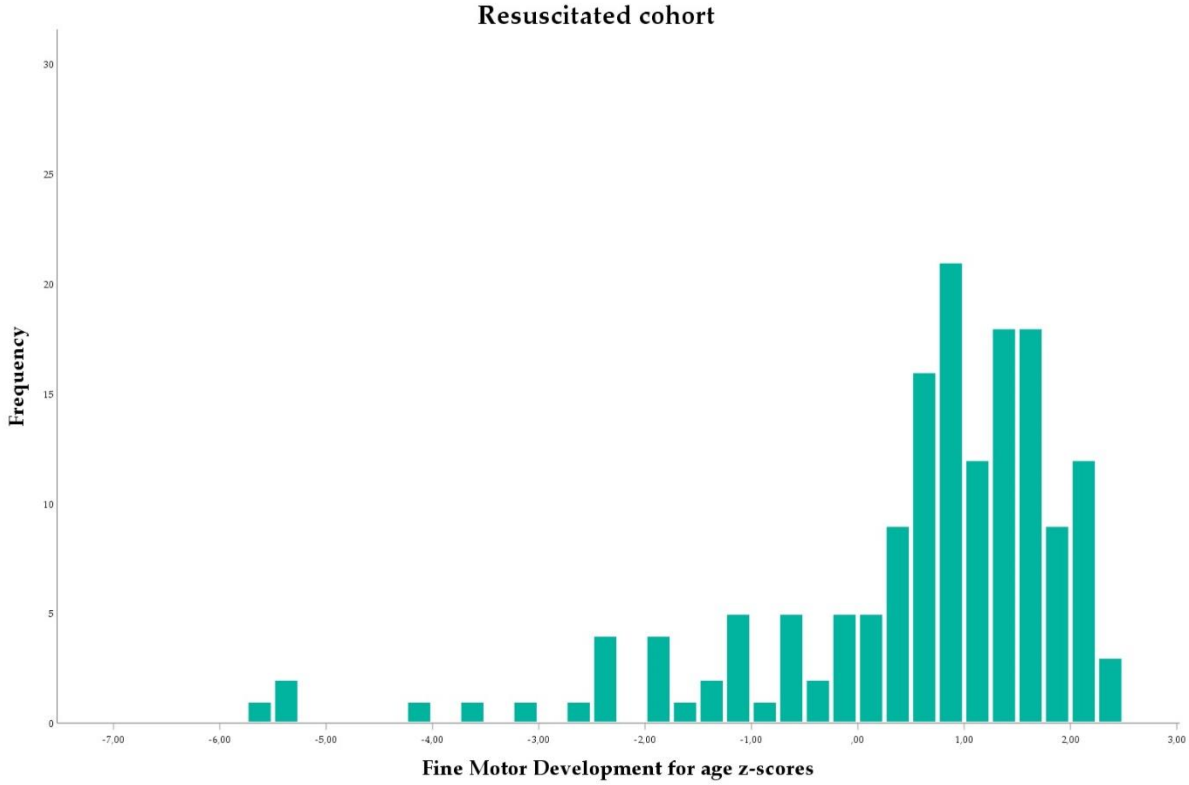

Figure S4

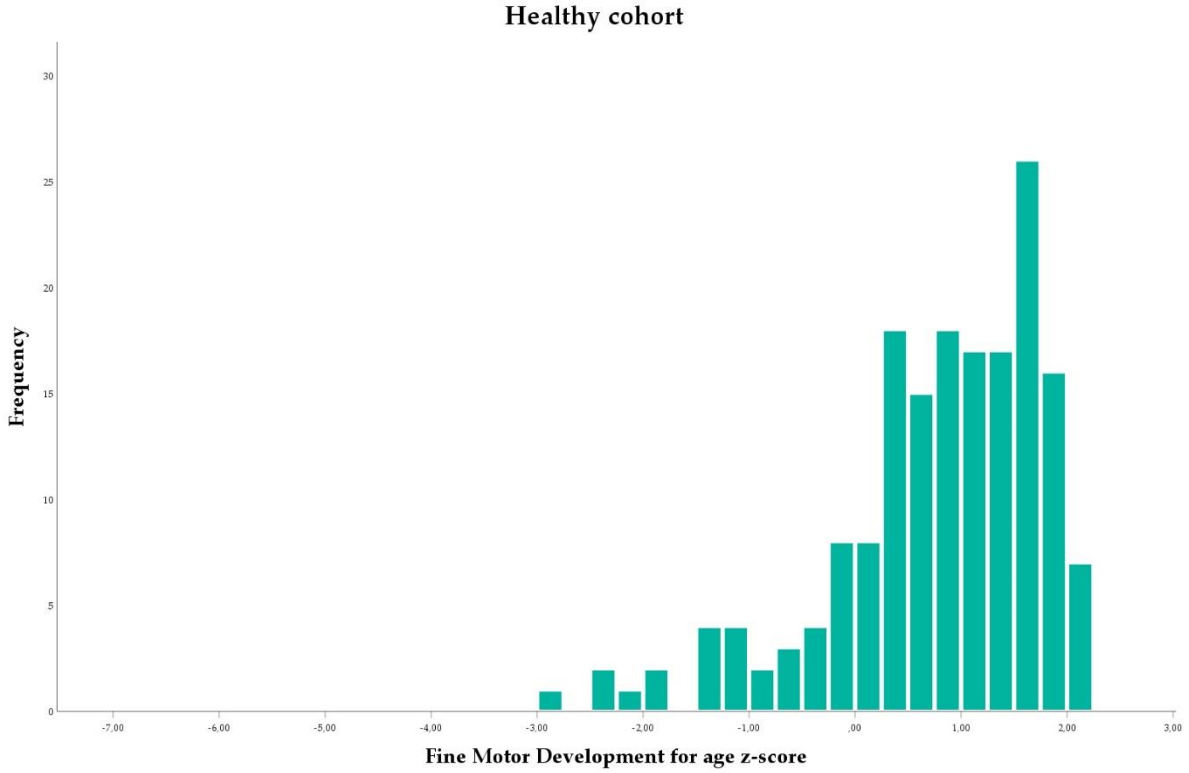

Figure S5

Figure S6

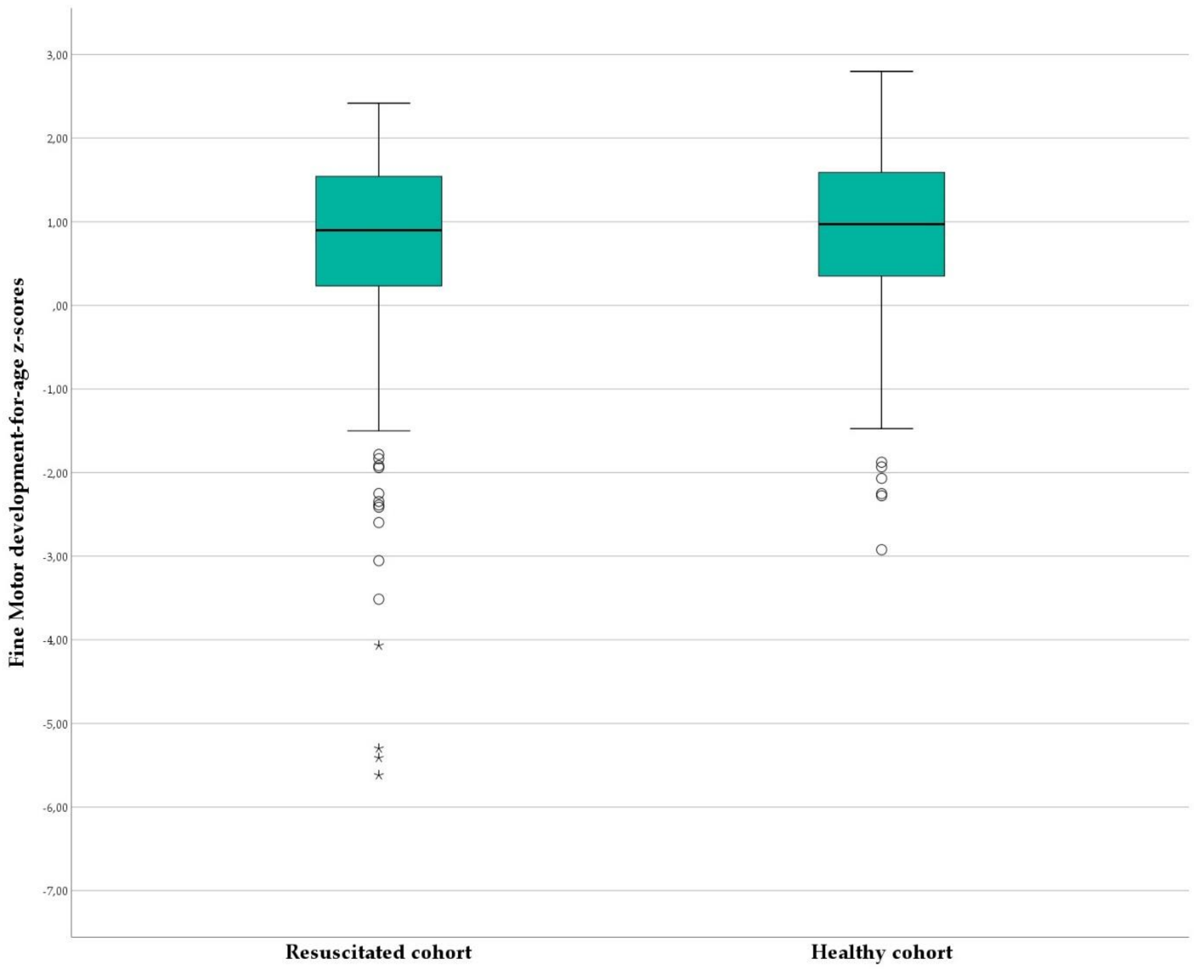

Fine Motor development-for-age z-scores
3,00
2,00
1,00
,00
-1,00
-2,00
-3,00
-4,00
-5,00
-6,00
-7,00
Resuscitated cohort
Healthy cohort

Figure S7 Language:

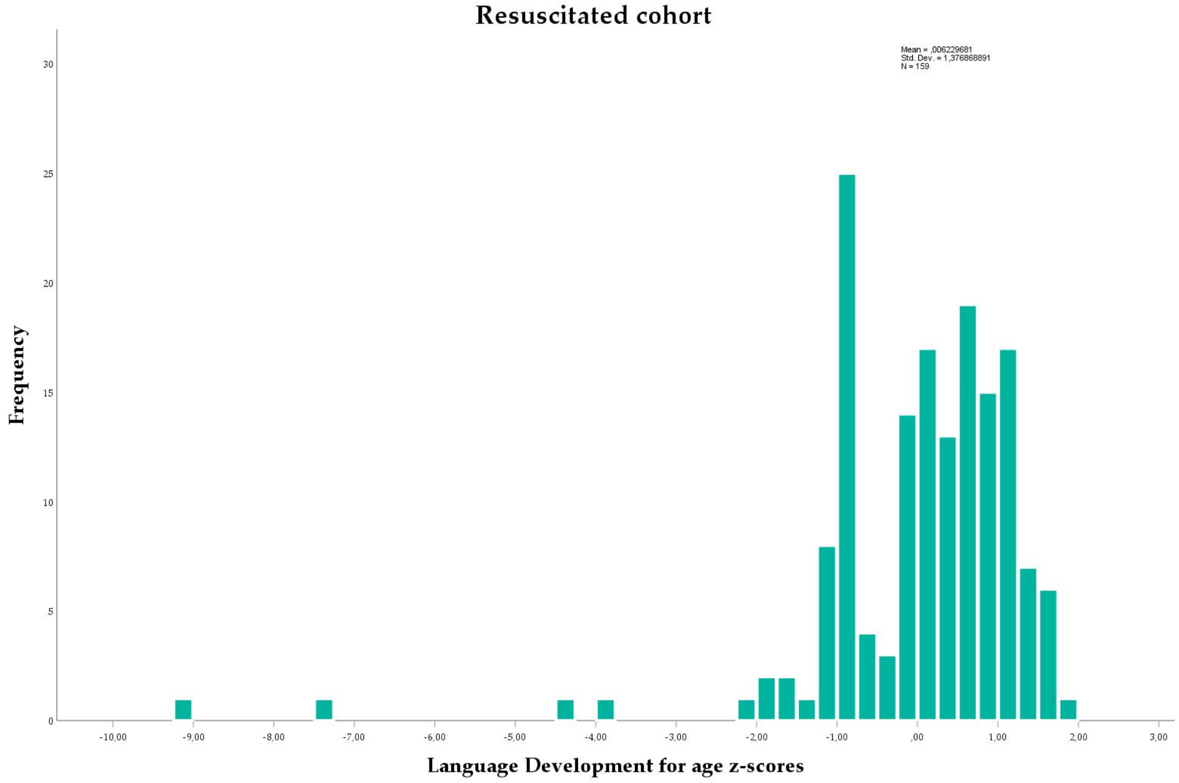

Figure S8

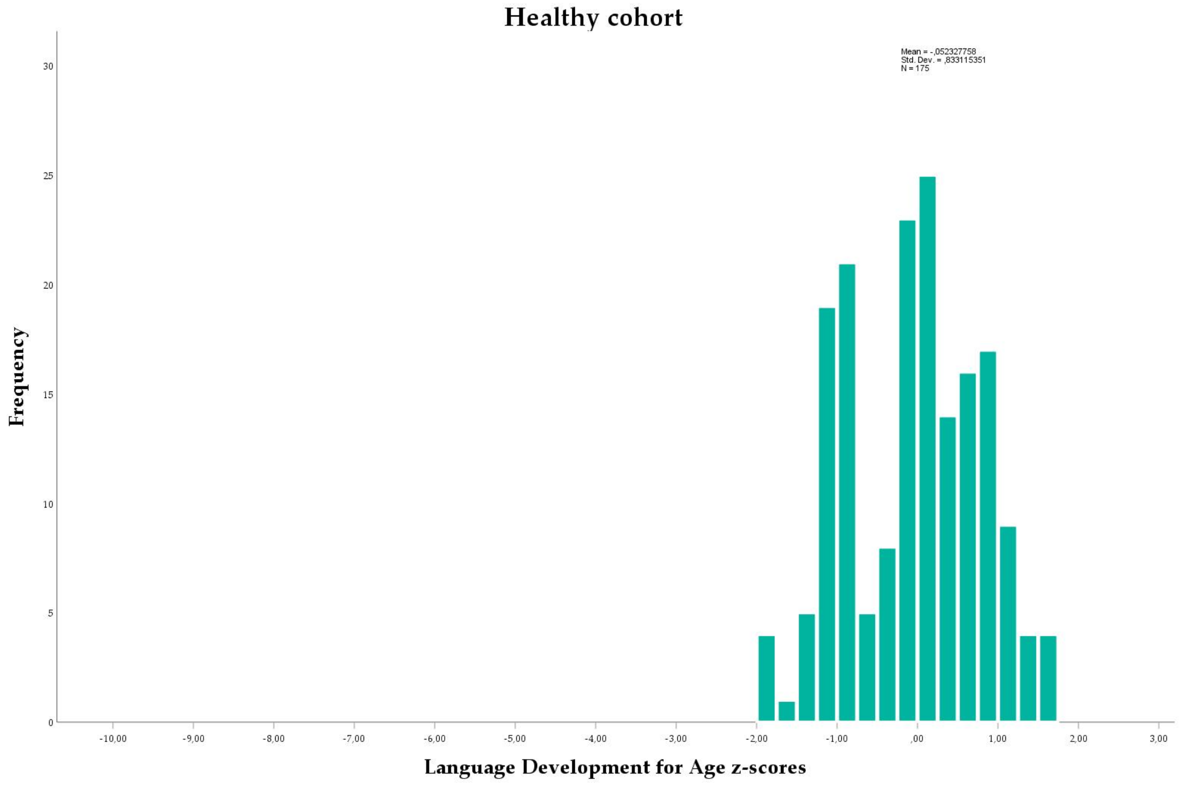

Figure S9

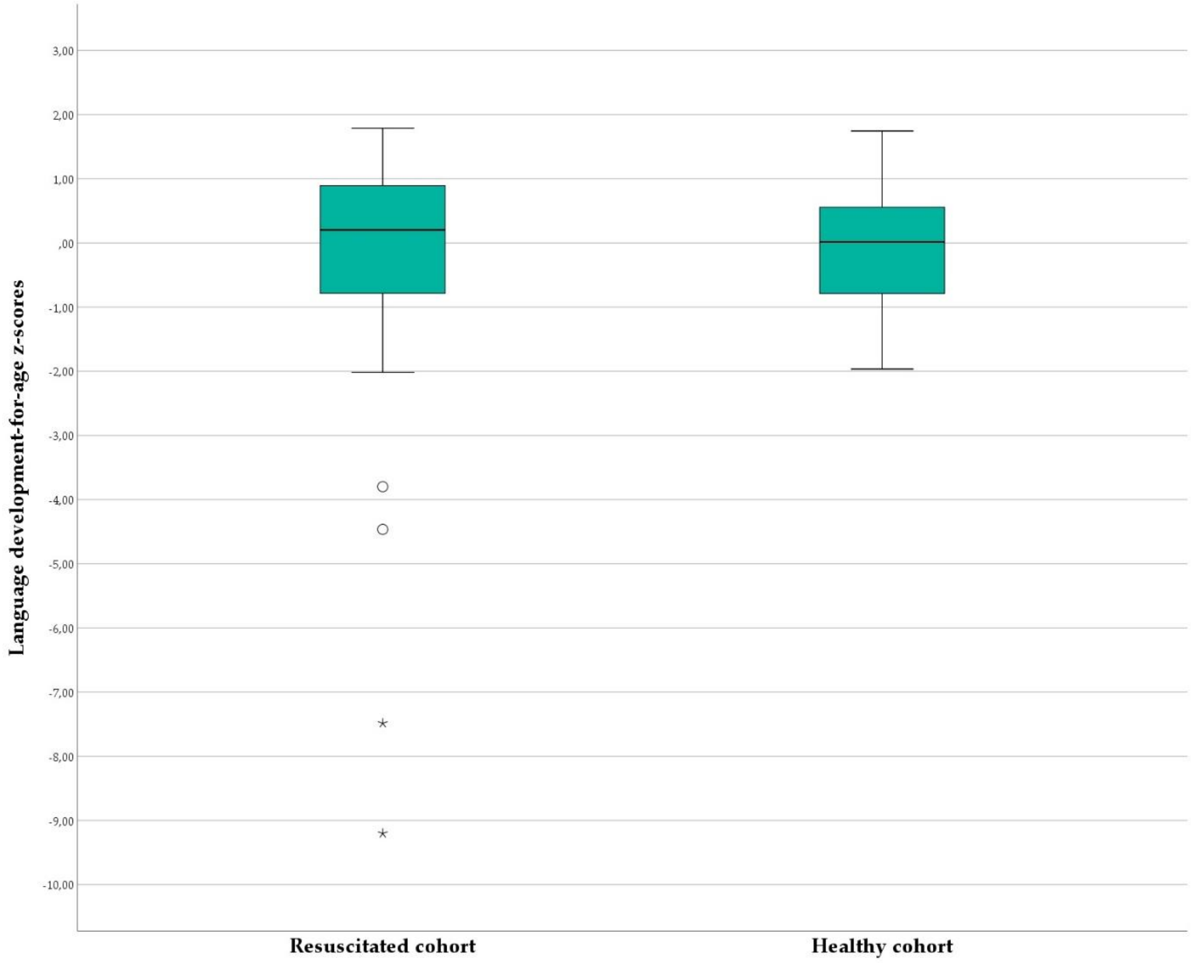

Figure S10 Social:

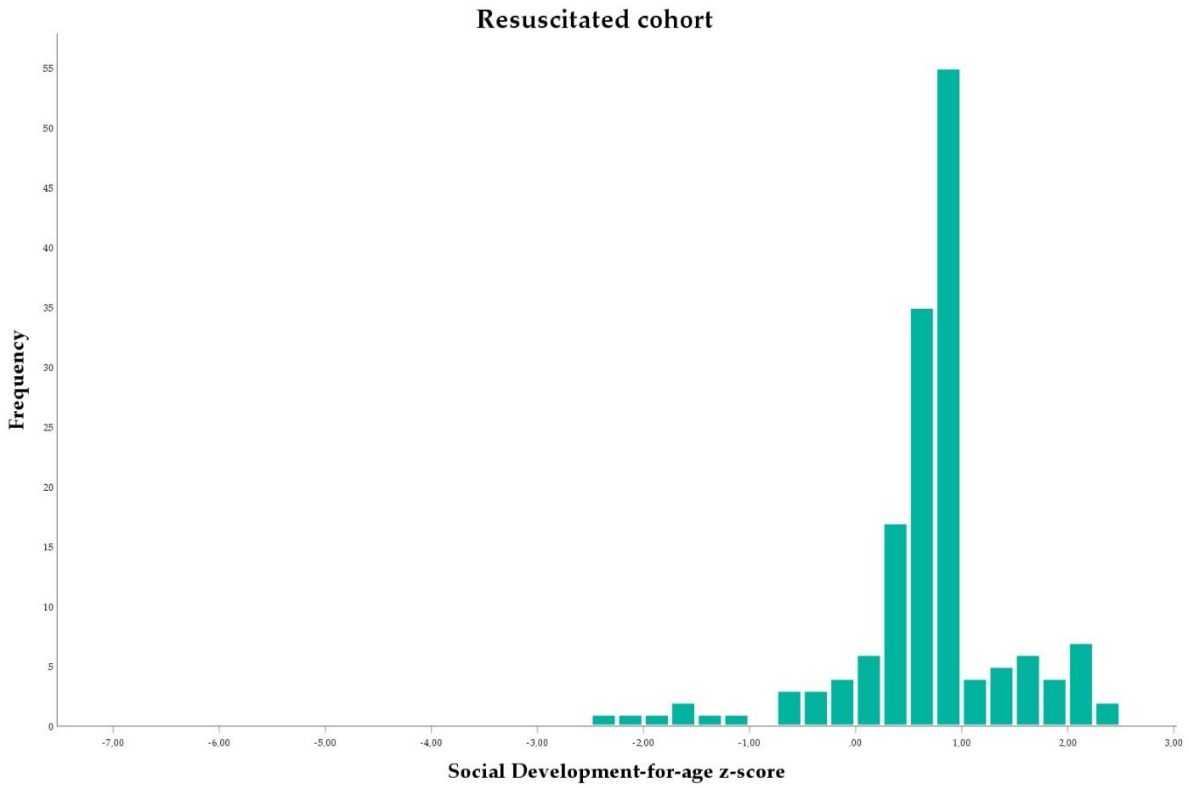
Resuscitated cohort
Frequency
Social Development-for-age z-score

Figure S11

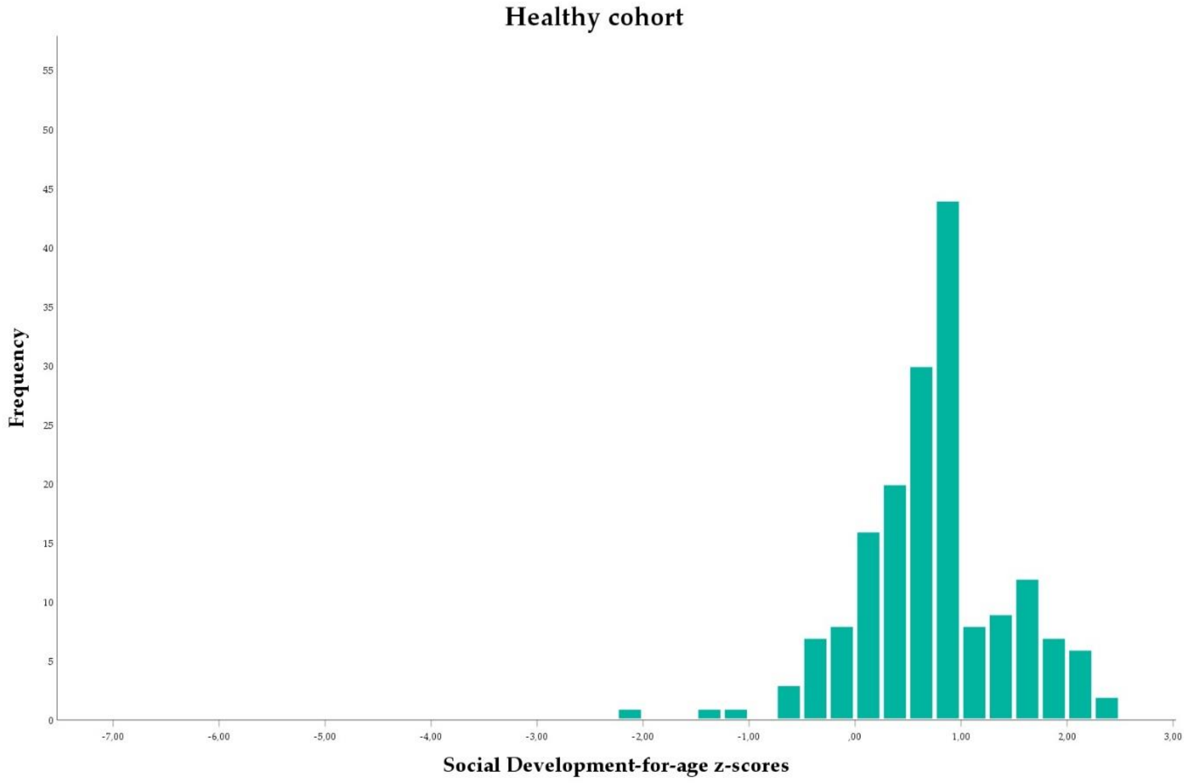
Healthy cohort
Frequency
Social Development-for-age z-scores

Figure S12

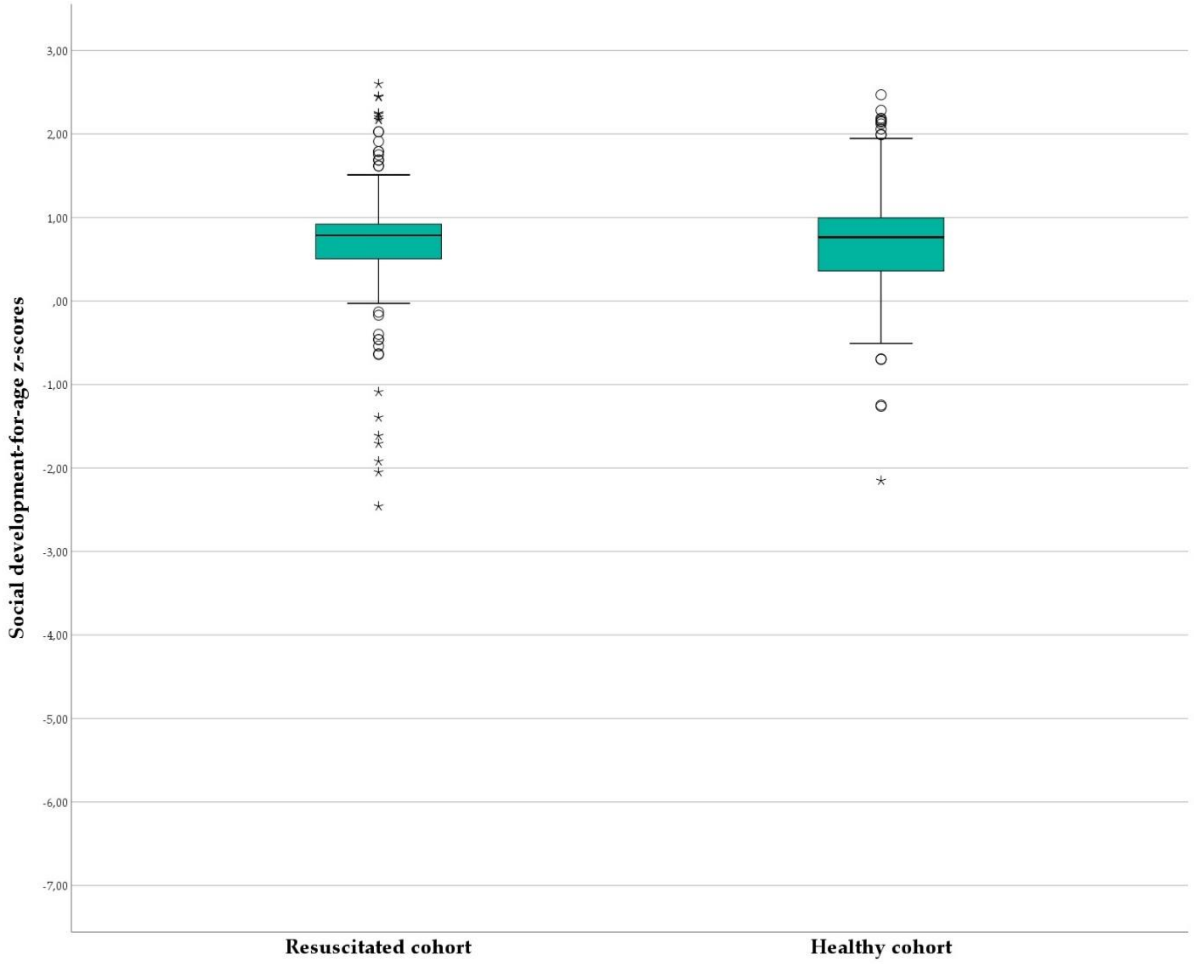
3,00
2,00
1,00
,00
-1,00
-2,00
-3,00
-4,00
-5,00
-6,00
-7,00
Social development-for-age z-scores
Resuscitated cohort
Healthy cohort
